# Microplastics and invasive crayfish: emerging interactions and ecological implications from three coexisting species in a subalpine lake

**DOI:** 10.1038/s41598-025-18595-6

**Published:** 2025-09-29

**Authors:** Giulia Cesarini, Marco Orlandi, Riccardo Sbarberi, Raffaella Sabatino, Stefano Magni, Andrea Binelli, Nicole Santi, Denise Schiavetta, Lyudmila Kamburska, Mirko Zanini, Silvia Zaupa, Angela Boggero

**Affiliations:** 1 National Research Council-Water Research Institute (CNR-IRSA), Corso Tonolli 50, Verbania Pallanza, 28922 Italy; 2 Department of Biosciences, University of Milan, Via Celoria 26, Milan, 20133 Italy; 3 National Biodiversity Future Center, NBFC, Piazza Marina 61, Palermo, 90133 Italy; 4 Maddalena & Associati Sagl, Via Ai Rïégn, Gordevio, 6672 Switzerland

**Keywords:** Plastic pollution, Alien species, Freshwater ecosystems, Microplastic removal, Bioindicators, Environmental monitoring, Invasive species, Limnology, Environmental impact, Environmental monitoring

## Abstract

Microplastics (MPs) and invasive species are two of the most pressing threats to freshwater ecosystems, yet their interactions remain underexplored. This study presents the first comparative analysis of MP uptake among three coexisting invasive crayfish species (*Faxonius limosus*, *Pacifastacus leniusculus*, and *Procambarus clarkii*) from Lake Maggiore, a large subalpine lake subjected to intense anthropogenic pressure. A total of 90 individuals were analyzed for biometric traits and MP occurrence in intestinal content, with species identification confirmed via molecular analysis. No significant interspecific differences or correlations with biometric traits were found, though *F. limosus* showed the highest average concentration. Most MPs were < 1 mm polyester or polyacrylate fibers, suggesting a dominant domestic source. Additionally, this work provides the first evidence of MP uptake in *F. limosus*, filling a key knowledge gap. Beyond documenting MP ingestion, our findings support the potential of invasive crayfish as agents of MP removal, suggesting an ecological role with important implications for environmental monitoring and ecosystem management. These results also highlight the need for further research on trophic transfer and organ-level accumulation of MPs, especially in widely distributed invasive species that are increasingly relevant for environmental risk assessment.

**Supplementary Information:**

The online version contains supplementary material available at 10.1038/s41598-025-18595-6.

## Introduction

Freshwater ecosystems, though covering a small portion of Earth’s surface, are highly biodiverse and provide vital ecological, economic, and cultural services^1^. However, they face increasing threats that jeopardize their biodiversity and functions^2^. Pollution from agricultural runoff, untreated sewage, industrial effluents, and plastic waste severely degrades freshwater quality and habitats^3,4^. Additionally, the introduction of invasive species, whether intentional or accidental, exacerbates these pressures by displacing native biota, altering trophic interactions, and facilitating the spread of pathogens^5^.

Plastic production has surged in recent years, driven by increasing global demand across various industries, including packaging, construction, and consumer goods^6^. In 2023 alone, global plastic production was estimated to exceed 413.8 million metric tons (Mt), reflecting a persistent upward trend^7^. Plastics, according to their sizes, are classified in macroplastics (> 5 mm), large microplastics (lMPs, 5 –1 mm), microplastics (MPs, 1 mm–1 μm) and nanoplastics (1 μm-100 nm)^8^. In this paper, the term “MPs” will be used to refer collectively to both large microplastics (lMPs, 5 –1 mm) and microplastics (MPs, 1 mm–1 μm), unless otherwise specified. MPs are classified as primary or secondary based on their origin: primary MPs result directly from industrial processes, such as plastic pellets^9^, while secondary MPs arise from the fragmentation of plastic debris or from textiles during washing and wear^10^. Notably, physicochemical variables can speed up the deterioration of plastics through mechanical stimulation, biological, thermal, and photo-oxidative degradation^11^.

Freshwater ecosystems are less studied in the context of plastic pollution compared to other environments, and significant knowledge gaps remain regarding the sources, pathways, fate, and distribution of plastics within them^12^. Aquatic biota plays a crucial role in the transport, temporary storage, and transformation of plastics in these ecosystems^13^. A broad variety of aquatic organisms, including birds^14^, fish^15^, bivalves^16^, crustaceans^17^ and other invertebrates ingest MPs leading to various toxic effects^18^. These include behavioural modification, metabolic disorders, and compromised immune responses, each of which poses significant threats to the composition and stability of aquatic ecosystems^18^. Identifying sentinel species for monitoring plastics’ occurrence and their effects on ecosystems is essential for understanding their environmental impact and developing management strategies, as shown by previous studies^12,16,19^.

Invasive species are a major driver of biodiversity loss globally, with invasive crayfish being among the most widely introduced species in freshwater ecosystems^20,21^. Europe currently hosts 12 invasive crayfish species, four of which — *Faxonius limosus* (Rafinesque, 1817), *Pacifastacus leniusculus* (Dana, 1852), *Procambarus clarkii* (Girard, 1852), and *Procambarus virginalis* Lyko, 2017 — are listed as species of Union Concern^22,23^. Originating from North America, they were introduced in Europe and respectively in Italy for bait, fish forage, and food purposes^24^. Three species - *F. limosus*,* P. clarkii*,* and P. leniusculu*s - already coexist in Lake Maggiore^24,25^. *Faxonius limosus* (the spiny-cheek crayfish) was firstly found in Italy in Lake Iseo^26,27^ and later in Lake Maggiore^25,28^. *Procambarus clarkii* (the red swamp crayfish) was introduced in Italy in 1989^29^. It thrives due to its rapid reproduction, high fecundity rates, opportunistic diet, and aggressive nature^30^. It also transmits the deadly crayfish plague (caused by *Aphanomyces astaci* Schikora, 1906), which poses a significant threat to native crayfish^31^. *Pacifastacus leniusculus* (the signal crayfish) was first recorded in Italy in the Adige River in 1997^32^ and in Lake Maggiore (Swiss side) in 2017^33^. Notwithstanding all of them are among the most invasive crayfish worldwide^20,34^, since they have a remarkable adaptability, allowing them to thrive in a wide range of environmental conditions and an exceptional degree of tolerance to environmental stresses, they are frequently used as bioindicators in aquatic ecosystems^19,35^.

Lake Maggiore, characterized by growing urbanization, industrial and touristic activities is an interesting survey area and becoming a hotspot of invasive crayfish. In response to the increasing concerns about the harmful impacts of MPs^36^ and invasive crayfish on freshwater ecosystems and human health^28^, the aim of our pilot study was to assess quantitatively and qualitatively the MPs content in the intestine of three invasive crayfish species whose coexistence is evident in the Swiss sector of Lake Maggiore^23,24^.

Specifically, our hypotheses are: *H1)* we predict that the three coexisting invasive crayfish species will show different MP contamination rates, reflecting potential differences due to interspecific interactions; *H2)* we expect a positive correlation between crayfish size and the size of MPs ingested, with larger species ingesting a broader range of MPs^19,37^; *H3)* we hypothesize that the three species will ingest different MP types, consistent with species-specific feeding habits and previously observed associations^38^.

## Materials and methods

### Study area and samplings

**Fig. 1:**
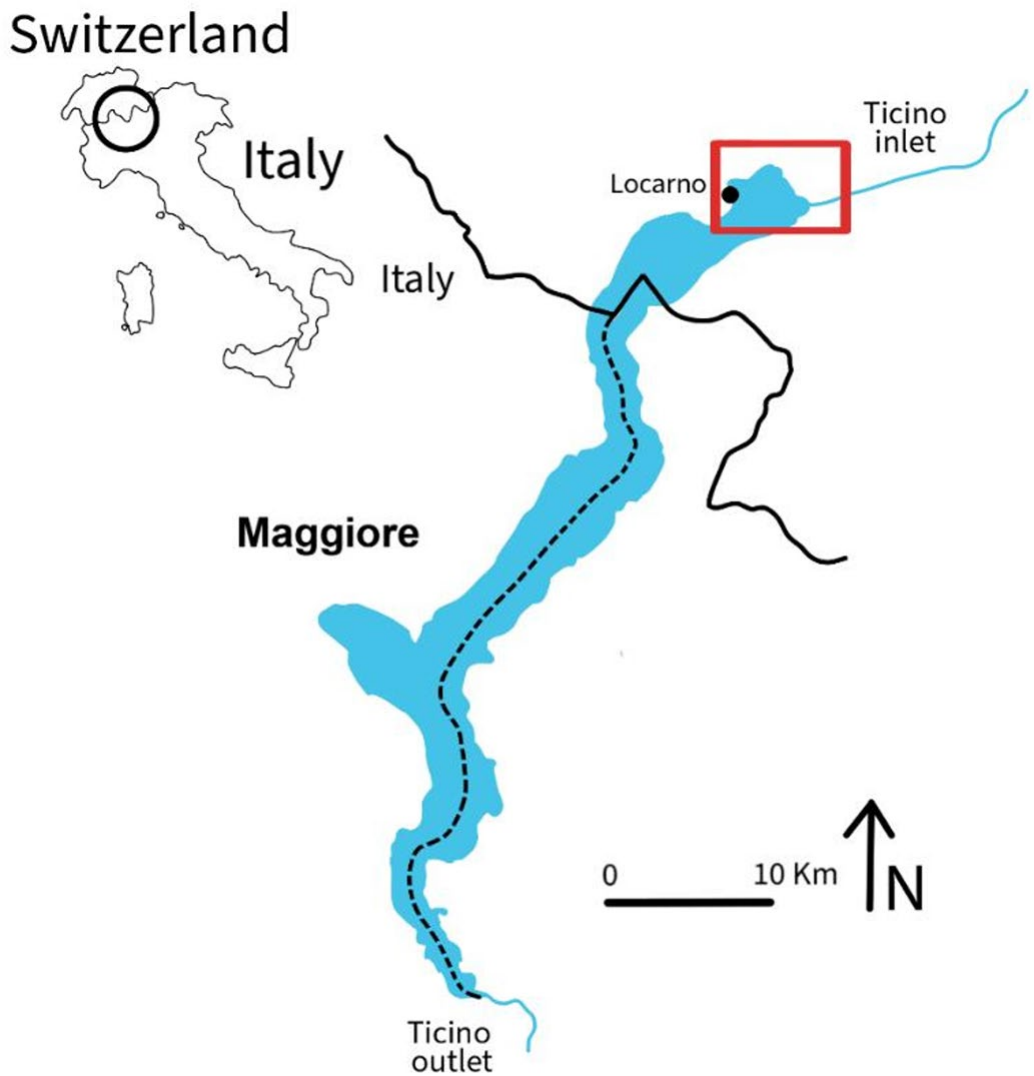
Map of the sampling area located in the Swiss sector of Lake Maggiore. Red polygon: area where invasive crayfish were taken. The map was manually drawn using Adobe Fresco (version 4.8.1) on an iPad 10th generation. Adobe Fresco is available at: https://www.adobe.com/products/fresco.html.

Sampling of invasive crayfish was carried out in Lake Maggiore (46° 5.88’ *N* − 08° 42.88’ E), one of the large and deep lakes of the Po Valley, in the north-western area (​​Piedmont, Italy). Only the Swiss part of the lake was considered, and only the Locarno surroundings (Fig. 1). Lake Maggiore, being included within the LTER *Subalpine lakes* macrosite (https://deims.org/8ffe6c61-5473-4e56-9a6e-827baad941e5), is regularly monitored for water chemistry and biology (mainly plankton and fish) within the International Commission for the Protection of Italian-Swiss Waters (CIPAIS) (www.cipais.org*)* since the 1980s^39^.

The study area extends from the town of Locarno (lakeside promenade) towards the Bolle di Magadino Nature Reserve, a protected area at the confluence of River Ticino into Lake Maggiore, up to Magadino municipality. From the shore of the lake to the inner part of the territory, the area extends to watercourses that connect the mainland to the River Ticino. From spring into early autumn, the Locarno Bay and lakeside promenade, surrounded by houses, to Tenero is bustling with tourists. Continuing towards Magadino, the Minusio and Magadino harbours and a large camping area characterised the lake littoral. On site trails allow the entrance to the Bolle di Magadino Nature Reserve (protected since the 1970s), known to be one of the last natural deltas in Switzerland, and representing a locally very popular Reserve.

For summer samplings, a total of 52 cylindrical flexible/collapsible nylon traps (30 × 60 cm) with double entrances and bait (ca. 20 catfood g/trap) were used, set al.ong the coast in the 0.5–3 m depth zone for 24 h. No native crayfish species inhabits the lake shores^24^.

The crustacean species studied in this work are not protected under European or national legislation and do not fall within the categories of homeothermic wild fauna (birds and mammals) or other protected species listed in Annex IV of Directive 92/43/EEC or Annex I of Directive 2009/147/EC. Consequently, ethical approval from an institutional committee was not required. All methods were carried out in accordance with relevant institutional and national guidelines and regulations. This study is reported in accordance with the ARRIVE guidelines (https://arriveguidelines.org).

After capture, traps were removed to avoid injuries and cannibalism among individuals, and to allow the release of by-catches of non-target species. Native species other than crayfish were released on site to safeguard local biodiversity. In the laboratory, crayfish were gradually anaesthetised and euthanized by exposure freezing temperatures (approximately − 20 °C), following a method commonly used in crustacean studies to minimize stress and avoid unnecessary suffering^19^.

### Biometric analysis

Invasive crayfish were later divided by sex, measured — total body length in mm (TL) and weight in g, cephalothorax length in mm (CL), both chelae length in mm and weight in g — and their species belonging was confirmed through complementary morphological analyses, using both naked eye examination and stereomicroscopy, alongside molecular analyses. A digital scale (Mettler Toledo PG 603-S: precision 0.001 g) and a digital calliper (Mitutoyo Digimatic: precision 0.1 mm) were used for biometric analyses.

A pool of 30 individuals per species was created after the captures to ensure a sufficient sample size to perform biological, chemical and statistical analyses. To create the pool, individuals of each species were chosen based on size: in detail, for the *F. limosus* pool, individuals larger than 4 cm (TL) were chosen, whereas for *P. leniusculus* and *P. clarkii*, individuals larger than 7 cm. Both male and female were included in each pool.

### Molecular analysis

To isolate and purify genomic DNA from tissue specimens, six individuals (3 males and 3 females) of each species of different size from the total pool of 30 individuals per species, were selected. The PureLinkTM Genomic DNA kit (Invitrogen, ThermoFisher Scientific) was used to extract DNA from samples, following the manufacturer’s instruction. For taxonomic identification, we targeted a fragment of the mitochondrial gene encoding for the cytochrome c oxidase subunit I (COI), using the invertebrate universal primers LCO1490 and HCO2198^40^. PCR assays were carried out in a reaction volume of 50 µl, applying protocols and conditions already described^41^. Amplification products were checked by agarose gel electrophoresis and sequenced by Sanger sequencing at Macrogen Europe (Milan, Italy). Electropherograms were checked in FinchTV 1.4.0 (https://digitalworldbiology.com/FinchTV). Forward and reverse sequences were trimmed and merged using Sequencher 5.0 (http://www.genecodes.com). Merged sequences were, then, blasted in GenBank to confirm the taxonomic identity of each sample (https://blast.ncbi.nlm.nih.gov/*)* and, once verified, deposited (Table S1).

### Microplastic analysis

To perform the digestion and MP content analyses, only the intestines of the crayfish previously frozen were preserved for examination. After defrosting, the posterior part of the digestive system was manually extracted by carefully removing the telson with the attached intestine, which was then weighed. The intestines of 10 individuals for each species (3 replicates, tot 30 individuals; *n* = 3 pools of 10 specimens) were pooled and homogenized, using a Potter-Elvehjem Tissue Homogenizer.

Homogenates were filtered using a vacuum pump on 8 μm cellulose nitrate membrane filters (Sartorius™ 50 mm). The filters were then treated with 15% hydrogen peroxide (H_2_O_2_) to complete the digestion of the organic residues left.

Suspected MPs were transferred from the filters to clean filters using tweezers and a stereomicroscope. The quantification and classification of each plastic particle in terms of shape, size, color, and polymer were carried out individually using the spectrometer µFTIR (Spotlight 200i equipped with Spectrum Two, PerkinElmer). The analysis was carried out using attenuated total reflectance (ATR) with 32 scans within the wavelength range of 600 to 4000 cm^− 1^ wavelength range. Spectra were compared with standard libraries (PerkinElmer) using the Spectrum 10 software. Following a visual examination of spectrum peaks by the operator, a matching score ≥ 0.70 was considered. MPs were categorised, based on their shape, in fibers, fragments, films, or pellets, and on their size^42^. To classify the MPs size was followed the ISO/TR 21,960 classification^8^. Although the µFTIR system allows the identification of particles larger than 10 μm, the visual sorting under a stereomicroscope limits the recovery of very small debris. In our samples, the smallest MP successfully identified had a maximum length of 0.16 mm (160 μm).

The quantity of synthetic particles was expressed both as number of items/individuals (items/ind.) and as items/g as mean value with standard deviation.

### Quality assurance/quality control (QA/QC)

All procedures were conducted under a laminar flow hood to minimize airborne plastic contamination. Glass equipment and Petri dishes were used exclusively, and filtered solutions (Milli Q water and H_2_O_2_) were employed throughout. Dissections were performed using sterilized steel scissors and tweezers, with researchers wearing cotton lab coats and nitrile gloves to ensure contamination-free handling.

To monitor the eventual atmospheric contamination by plastics (especially fibers), nitrate cellulose membrane filters (blanks), identical to those used for the samples, with a pore size of 8 μm, were processed in parallel to samples during homogenization and filtration. No plastics were detected in the blanks of *F. limosus* and *P. leniusculus*. In contrast, two polyester fibers (one black and one transparent) were found in the blank filter of *P. clarkii*. As two black polyester fibers were also detected in the *P. clarkii* samples, these were subtracted from the final count^42^.

### Statistical analysis

For each dataset the normality was assessed by Shapiro-Wilk test and the homoscedasticity was evaluated by Levene’s test. When the test indicated a deviation from normality, a logarithmic transformation was applied.

Then, to verify for significant differences within cephalothorax length (CL), total body length (TL) and weight (TW) across the three crayfish species, a one-way Analysis of Variance (ANOVA) was performed followed by Dunnett’s *post-hoc* test. The same analysis was also applied to evaluate potential differences in MPs concentration and size across the three species.

Regression analysis was performed for both sexes pooled to determine the relationship between total body length (TL in cm) and weight (TW in g) for each specimen of each species and commonly considered in crayfish instead of age determination^43^. Given that total body weight correlates with intestinal weight and total body length correlates with cephalothorax length, Pearson’s correlation test was performed only on intestinal weight and cephalothorax length to examine the relationship between the biometric features of crayfish and the MP characteristics found in their intestines (MP concentration: as items/ind. and as items/g, and MP size).

A Permutational Multivariate Analysis of Variance (PERMANOVA) using distance matrices (Adonis function in R) was applied to test for differences in shape, color and polymer composition across crayfish species.

The statistical significance level was set at *p* < 0.05. Statistical analyses were performed using GraphPad Prism software (version 8.0.1) and R version 4.3.1 (2023-06-16 ucrt).

## Results

### Species identification/confirmation

Molecular analyses confirmed the classification of the specimens. Specifically, three males and three females per species from the pool of 30 individuals were used for molecular investigations. All the blasted sequences exhibited a query coverage of 100.0%, an e-value of 0.0, and an identity percentage of 100.0%, ensuring the reliability of the molecular data.

### Biometric features of crayfishes

**Fig. 2:**
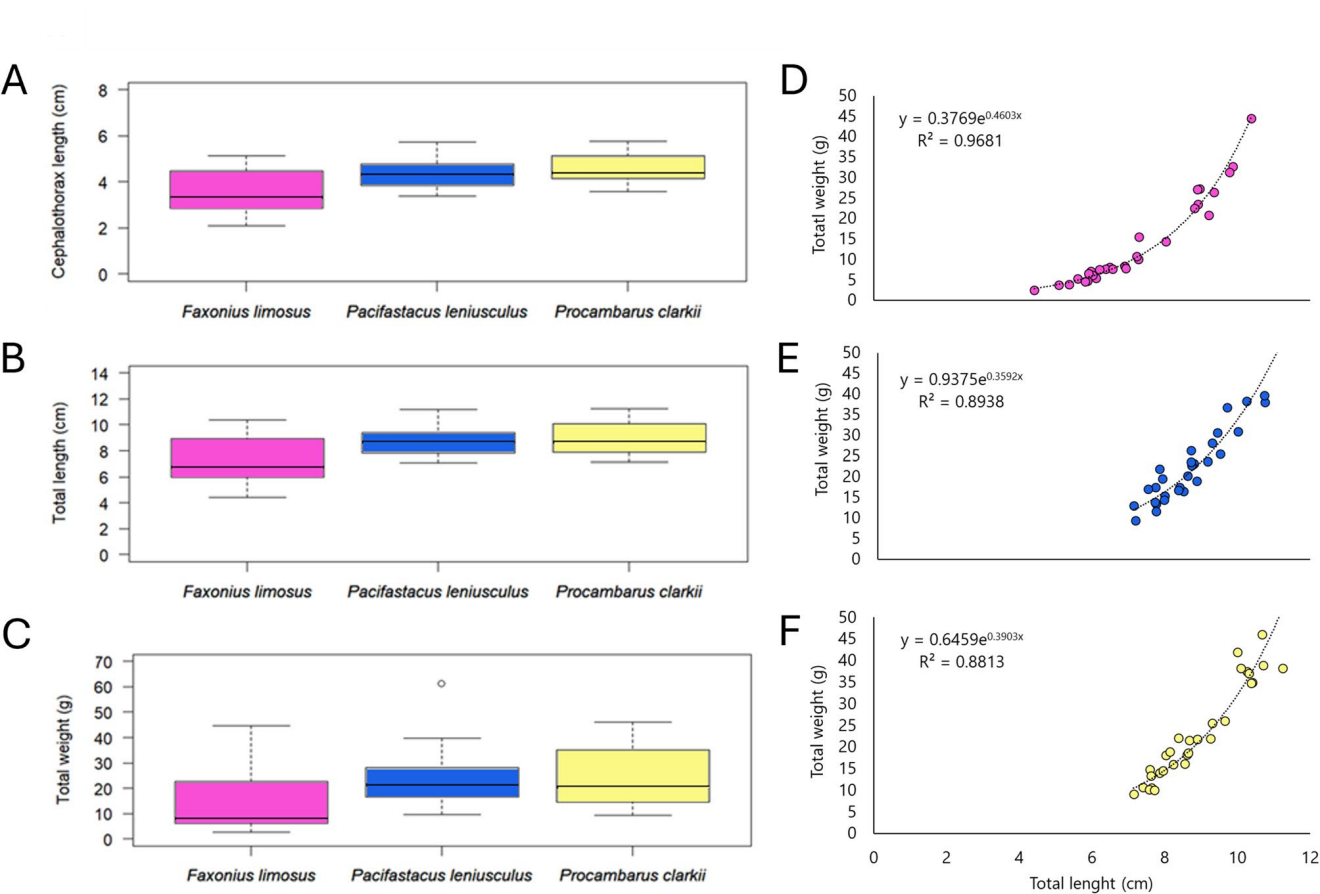
Boxplots of (**A**) cephalothorax lengths, (**B**) total body lengths and (**C**) total weights of the three invasive crayfish. Growth curves of the three invasive crayfish (**D**) *Faxonius limosus*, (**E**) *Pacifastacus leniusculus*, and (**F**) *Procambarus clarkii.* The x-axis label is the same in (**A**–**C**) and (**D**–**F**).

The total number of the three invasive crayfish caught during the sampling period was 90, divided into 30 individuals for each of the three invasive crayfish species. A summary of body size ranges is provided in Fig. 2 (A-C) and features in Table S2, where a larger size variability is observed for *F. limosus* compared to the other two species. The regression analysis of weight vs. total length yielded regression coefficients indicating positive allometric growth in all three invasive species (Fig. 2D-F).

*Procambarus clarkii* and *P. leniusculus* reached greater sizes and weights than *F. limosus.* Most specimens (67%) of *F. limosus* showed a size range between 5 and 8 cm, and a total average weight of 13.7 g, while most individuals of the other two species (77% of *P. clarkii* and 87% of *P. leniusculus*) show a size range between 7 and 10 cm and a total average weight of about 23 g. Maximum body size is 10.4 cm for *F. limosus*, and about 11.2 cm for *P. clarkii* and *P. leniusculus.* CL showed a size range between 2 and 4 cm for *F. limosus* (average length = 3.57 cm) and a similar dimensional range (3–5 cm) for *P. clarkii* (average length = 4.54 cm) and *P. leniusculus* (average length = 4.37 cm). Maximum body weight is 44.6 g (*F. limosus*), 46.1 g (*P. clarkii*) and 61.14 g (*P. leniusculus*), respectively. Subsequently, an ANOVA analysis was conducted to verify for significant differences within CL, TL and TW across the three crayfish species. Statistical significance was found for each morphological feature (CL: *f* = 16.83, *df* = 2, *p* < 0.001; LT: *f* = 15.14, *df* = 2, *p* < 0.001; TW: *f* = 7.87, *df* = 2, *p* < 0.001). Specifically, Dunnett’s *post-hoc* test detected significant differences between *F. limosus* and the other two species (CL: *F. limosus* vs. *P. clarkii*, *p* = 2.91 × 10^− 5^, and *F. limosus* vs. *P. leniusculus*, *p* < 0.001; LT: *F. limosus* vs. *P. clarkii*, *p* < 0.001, and *F. limosus* vs. *P. leniusculus*, *p* < 0.001; TW: *F. limosus* vs. *P. clarkii*, *p* < 0.001, and *F. limosus* vs. *P. leniusculus*, *p* < 0.001).

Regression analysis of total body weight vs. length revealed positive allometric growth across all three species. Notably, the weight-length relationship showed a more pronounced allometric growth pattern in *P. clarkii* and *P. leniusculus*, suggesting that weight increased at a significantly faster rate than body length in these species (Fig. 2E-F).

### Microplastics contamination in invasive crayfish

**Fig. 3:**
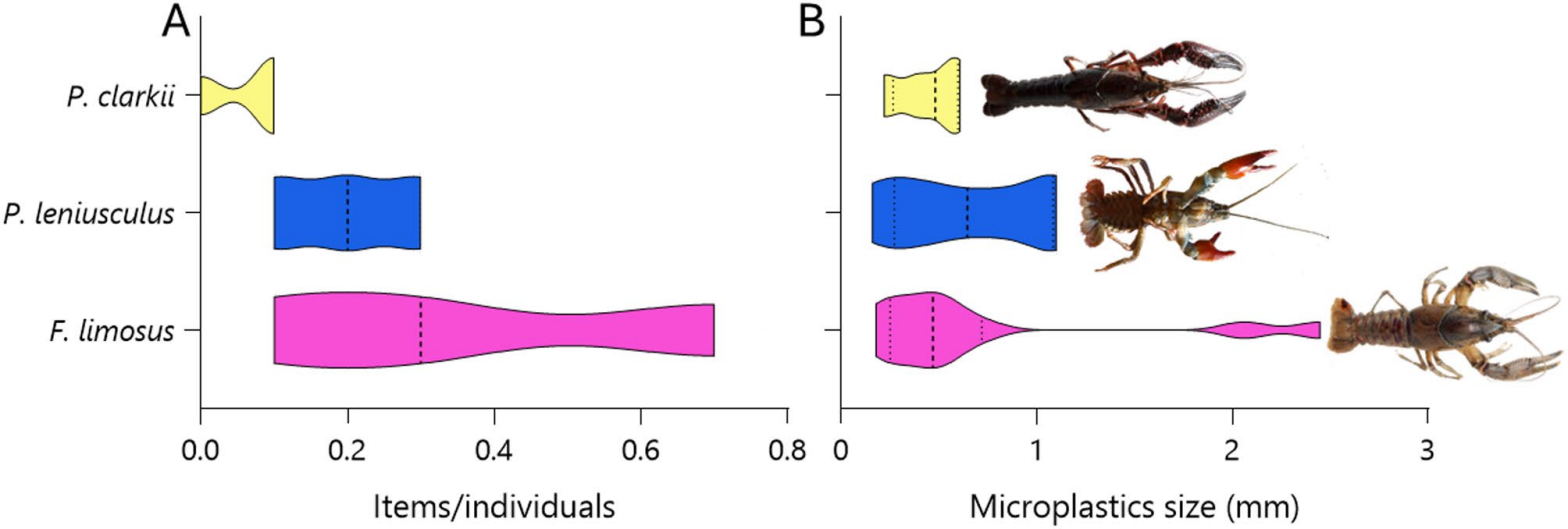
(**A**) Number of MPs in the intestine of the three invasive crayfish, and (**B**) their size distribution.

In this pilot study, the three invasive species found in the same area of Lake Maggiore exhibited mean MP concentrations in their intestine of 0.37 ± 0.31 items/ind. corresponding to 6.13 ± 4.01 items/g in *F. limosus*, 0.17 ± 0.10 items/ind. (2.17 ± 1.22 items/g) in *P. leniusculus*, and 0.07 ± 0.06 items/ind. (0.91 ± 0.79 items/g) in *P. clarkii* (Fig. 3A). Comparing MP concentrations across the three species, no significant statistical differences were found (ANOVA: *f* = 2.1, *df* = 2, *p* = 0.204). MP occurrence (Table S3) was 3/3 pools (100%) for *F. limosus*, 3/3 pools (100%) for *P. leniusculus*, and 2/3 pools (67%) for *P. clarkii*.

Regarding MP sizes, they were in the range 0.18–2.45 mm (average 0.54 mm) in *F. limosus*, 0.16–1.10 mm (average 0.55 mm) in *P. leniusculus*, and 0.22–0.61 mm (average 0.41 mm) in *P. clarkii* (Fig. 3B). No significant differences were observed within MPs sizes across the three species (ANOVA: *f* = 0.112, *df* = 2, *p* = 0.897). More in detail, according to ISO/TR 21,960 classification, two MP size ranges were identified: 1 mm–1 μm and 5 –1 mm. The relative proportions were 82% and 18% in *F. limosus*, 67% and 33% in *P. leniusculus*, while 100% *P. clarkii* showed exclusively the smaller size range. All the characteristics of MPs were shown in Table S3, while in Figure S1 and S2 are respectively reported the main obtained MP spectra and the raw µFTIR images of particles.

**Fig. 4:**
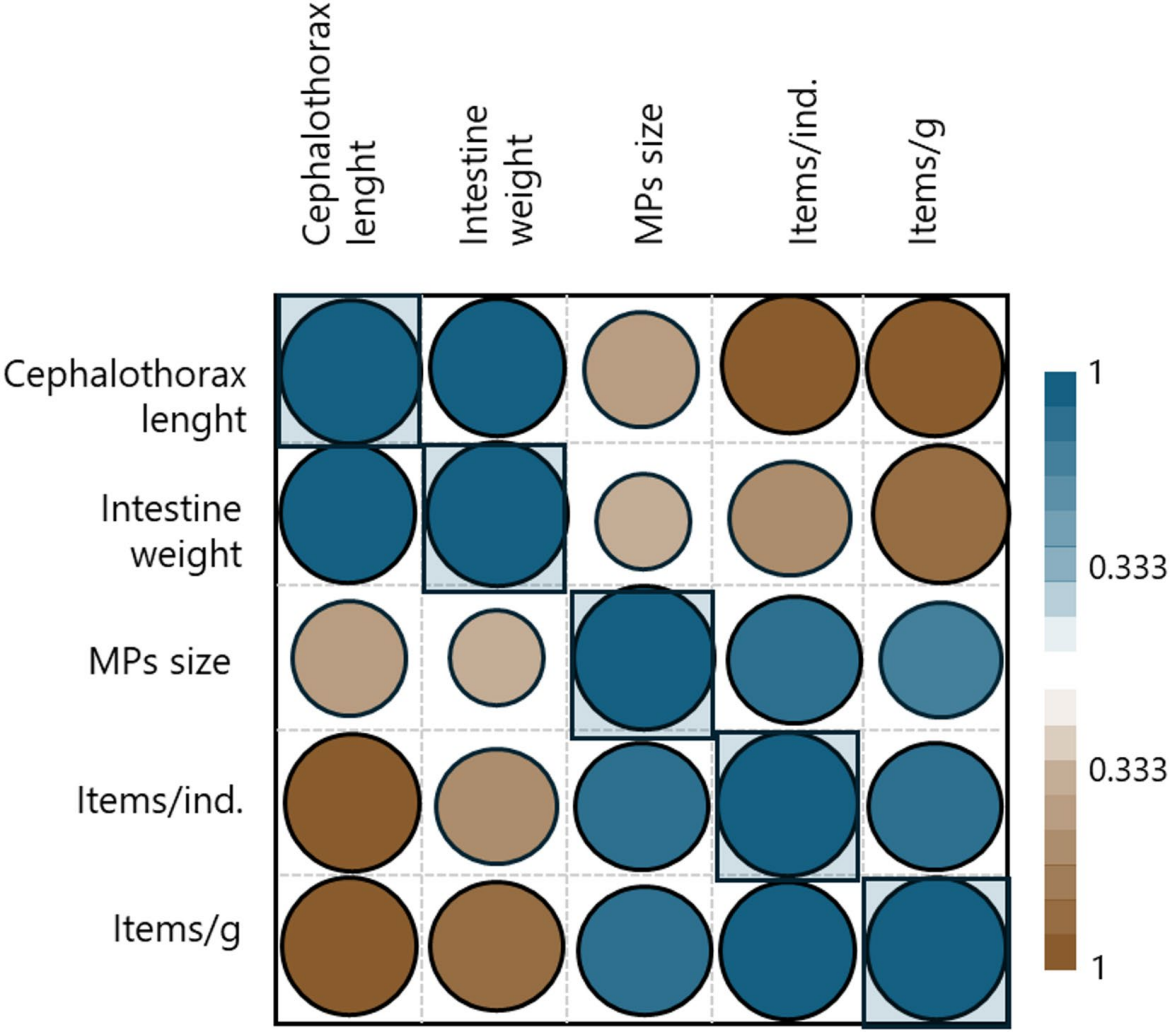
Pearson’s correlation test for cephalothorax length and intestine weight vs. microplastic features (number of items/ind., number of items/g and MP size). Significant results (*p* < 0.05) are boxed.

Pearson’s correlation test showed no significant correlations between the biometric features of invasive crayfish vs. MP features (size and number of items) (Fig. 4).

**Fig. 5:**
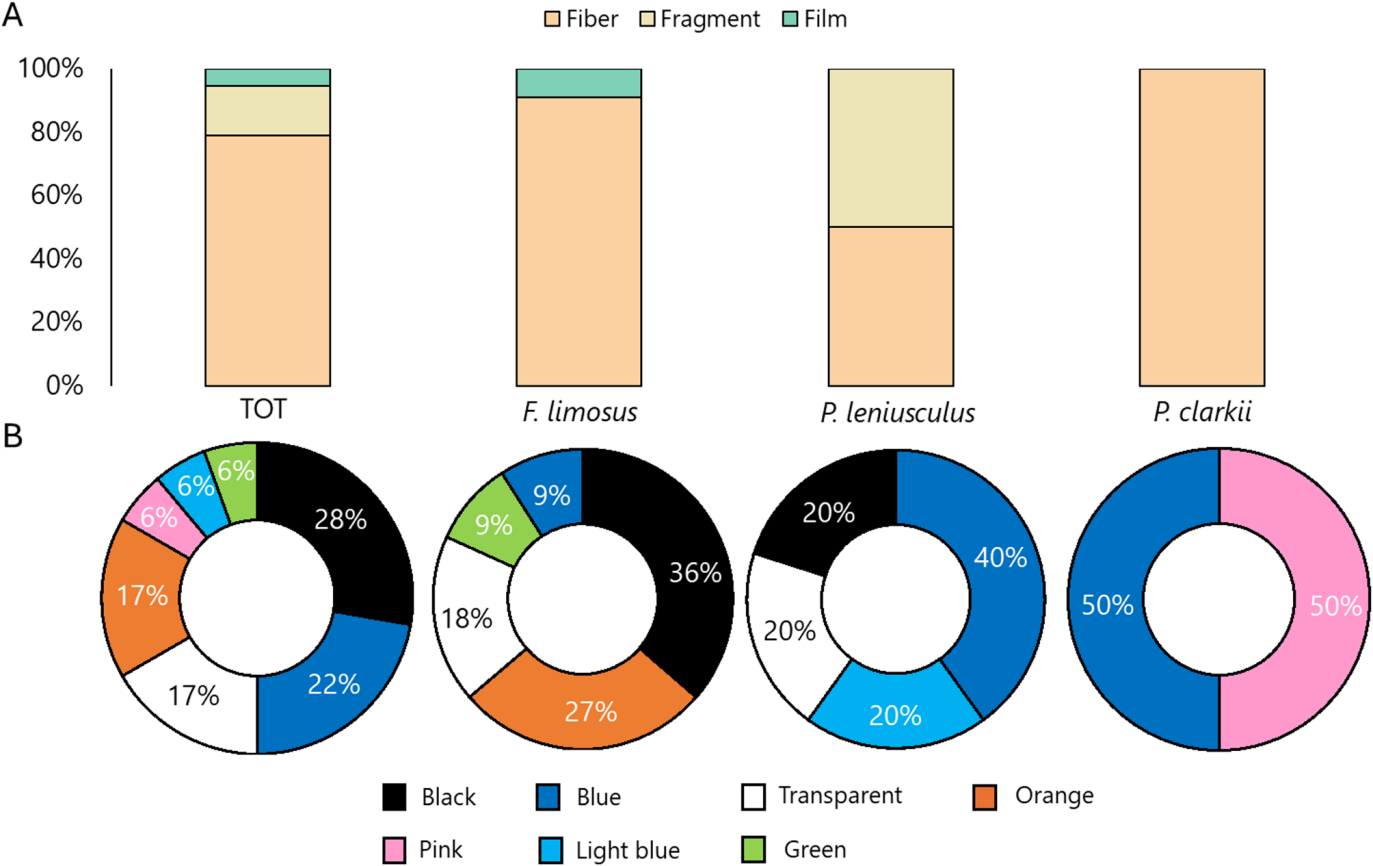
% MP (**A**) shape and (**B**) color found in the intestine of the invasive crayfish showed as total and per species.

Overall, fibers were the most prevalent shape, accounting for 79% of the total, followed by fragments (16%) and films (5%). Specifically, *F. limosus* showed 91% MPs as fibers and 9% as films (Fig. 5A). *Pacifastcus leniusculus* displayed both fibers and fragments as equally represented, whereas in *P. clarkii* 100% MPs were fibers. MP shape did not show significant differences across the three species (Adonis-PERMANOVA: R^2^ = 0.273, *p* = 0.064).

Analyzing color distribution, overall, black emerged as the most common color accounting for 28% of the total, followed by blue (22%) (Fig. 5B). Both transparent and orange colors tied at 17% each, while pink, light blue, and green each comprised 6% of the total. In *F. limosus*, black (36%) was the main color, followed by orange (27%), transparent (18%), blue and green (9% each). In *P. leniusculus*, blue was the most common color (40%), and the others were light blue (20%), transparent (20%), and black (20%). In *P. clarkii*, MPs were only blue and pink, with each accounting for 50%. Color did not show any significant difference across the three species (Adonis: R^2^ = 3.011, *p* = 0.060).

**Fig. 6:**
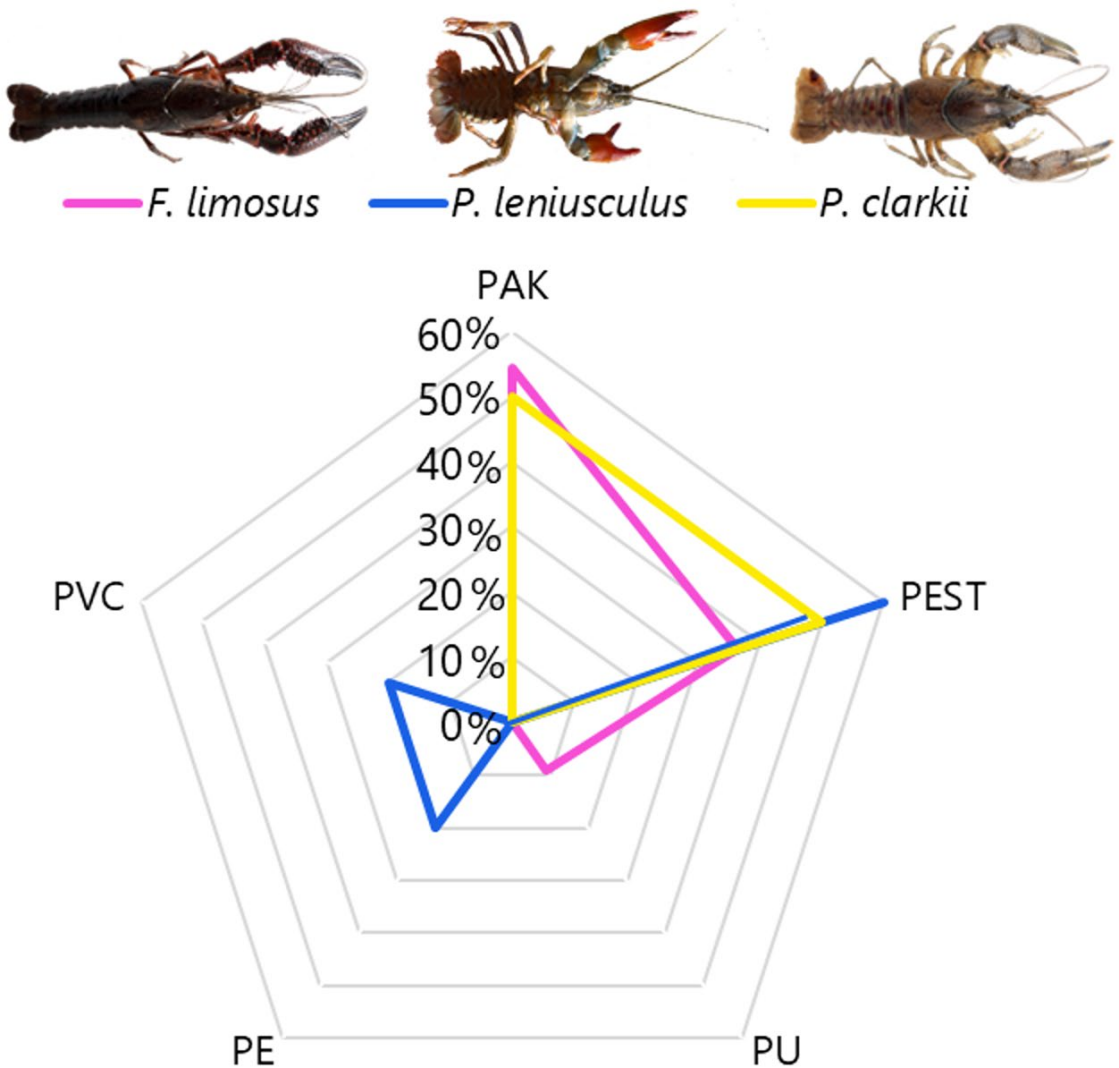
% MP polymer type found in the intestine of the three invasive crayfish and as total amount. Polyacrylate (PAK), polyester (PEST), polyurethane (PU), polyethylene (PE), polyvinyl chloride (PVC).

Concerning polymers, overall, polyester (PEST) was the most frequent accounting for 44%, followed by polyacrylate (PAK) with 39%, polyurethane (PU), polyethylene (PE), polyvinyl chloride (PVC) each 6%. Specifically in *F. limosus*, PAK represented the 55% of MPs, PEST the 36% and PU the 9% (Fig. 6). In *P. leniusculus*, PEST was the 60%, followed by PE and PVC, which each contributed for 20%. In *P. clarkii*, PAK and PEST each account for 50%. Polymer composition did not differ significantly across the three species (Adonis: R^2^ = 1.188, *p* = 0.338).

## Discussion

Lake Maggiore, one of the largest and deepest subalpine Italian lakes, is known for its floating plastic contamination ranged from a minimum of 0.02 plastics/m³ in September to a maximum of 0.29 plastics/m³ in December^36^. Our results suggest the lake as an essential model area for understanding the impact of multiple environmental pressures, especially for MP contamination, due to its capacity to collect pollutants from a vast surrounding basin. MPs in freshwaters come from various sources, including plastic bag fragments, ropes, and synthetic textile fibers. The increasing interest in MPs’ pollution highlights the crucial role plays by lakes as sink for plastics^12,44^. In this study, we found that all three invasive crayfish species (*F. limosus*, *P. leniusculus*, and *P. clarkii*) ingested MPs, with *F. limosus* showing the highest average concentrations, while no significant differences were observed in type, color, or shape of MPs among species.

Invasive crayfish have recently attracted attention for their potential role in monitoring MP pollution, particularly due to their benthic habits and wide distribution^19,45^. Evidence from Lake Maggiore further underscores the complex interactions between invasive crayfish and MP contamination in freshwater systems^28,36^. However, this study did not aim to evaluate their ecological indicator value or assess MP levels in surrounding abiotic compartments. Instead, our objective was to perform a comparative analysis of MP ingestion among three coexisting invasive species, providing new insights into species-specific exposure and polymer composition within their intestinal content.

### Biometric characteristics and competitive interaction

All three invasive crayfish species were found in close proximity within the same stretch of coast. To the best of our knowledge, this coexistence appears to be quite uncommon. Studies on the coexistence of invasive crayfish in some aquatic ecosystems have already been reported^46–48^. However, the coexistence of three invasive crayfish species has rarely been reported^49^, probably because the presence of *P. clarkii* seemed to affect the feeding habits and trophic niches of other two invasive crayfish, *P. virginalis* and *F. limosus. Procambarus clarkii* has been found closer to the watercourse entering Lake Maggiore (Magadino channel), while the other species predominantly inhabit the lake shore area. The niche separation arises from *P. clarkii* attitude to compete for space and resources against the other two species^48,49^ thanks to more rapid growth and sexual maturation, greater fecundity and breeding frequency, and tolerance of warmer or more degraded habitats^50^.

It is widely recognized that *P. clarkii* and *P. leniusculus* reached larger sizes compared to *F. limosus*^24,25^. Adults of the former two species typically range from 5.5 to 15 cm in size, whereas *F. limosus* rarely exceeds 10 cm in nature, in contrast with the research by^51^ where the species can grow beyond 10 cm in length due to its strong adaptation to the prevalent environmental conditions in Estonia. Our biometric data are consistent with those already published in previous studies^24,48,52^ confirming the intrinsic differences in size between the three species. Nevertheless, the smaller size attained by *F. limosus* could also be due to increased intraspecific competition with the other two crayfish species, as shown by several authors^53,54^. *Pacifastacus leniusculus* and *P. clarkii* have a strong ability to outcompete *F. limosus* in mixed populations^55^. At the same time, invasive crayfish constitutes a food source for various native and invasive fish and birds^56,57^. This predator-prey interaction may significantly influence their growth, demographic patterns, and the overall community composition and behavior^25,26^.

### State of the Art and quantification of MPs in invasive crayfish

This study marks the first presentation of comparative results on MP uptake across three coexisting invasive crayfish. Most notably, it also represents the first report of MP uptake in *F. limosus*. To date, research on MP ingestion in invasive crayfish is limited, with most studies focusing predominantly on *P. clarkii*, while no data were reported before on *F. limosus* (Table 1). For other invasive crayfish, there is only a single study on *Faxonius cristavarius* Taylor, 2000^58^. This knowledge gap underscores the importance of broadening research efforts to better understand interspecific differences in MP accumulation across species and different environments.

**Table 1:** Characteristics of MPs found in invasive crayfish from the scientific literature (ordered by year of publication).

Species	MPs abundance	MPs size	MPs shape	MPs color	MPs polymer	Identification method	Study site	Reference
*P. clarkii*	2.5 ± 0.6 items/ind.	< 1 mm	Fibers	White	PE, (PP)	µ-FTIR	Rice-Fish Co-culture, Shanghai, China	Lv et al., 2019^37^
*P. clarkii*	0.17 ± 0.07–0.92 ± 0.19 items/ind.	50–100 μm	Fibers, fragments	Red, transparent, (Blue)	PE, PP: PE	µ-FTIR	Pond and Rice-Crayfish Co-culture, Hubei, China	Zhang et al., 2021^59^
*P. clarkii*	0.86 ± 0.76 items/ind.; 0.06 ± 0.06 items/g (2021), 0.66 ± 0.76 items/ind.; 0.045 ± 0.06 items/g (2022)	134–1505 μm	Fibers, (fragments)	Black, white, (blue, light blue)	PP, PET	µ-FTIR	Lake Candia, Piedmont, Italy	Pastorino et al., 2023^19^
*P. leniusculus*	16.1 ± 6.9 items/ind.; 23.0 ± 11.4 items/g	<5 mm (not specified)	Fibers, (fragments, films)	White, (black, blue, red, green, yellow, purple)	EP, PE, PEST	µ-FTIR	Watercourses within the Yorkshire Dales National Park, North Yorkshire, England	Dent et al., 2023^38^
*P. leniusculus*	1–2 items/ind.	70–450 μm	Fibers, (fragments, films)	Black, (blue, red, pink, transparent)	PTFE, (PP, PE, PMMA, Cellophane, PA)	µ-FTIR	Wieprza River and its tributary, Poland	Dobrzycka-Krahel et al., 2024^60^
*F. cristavarius*	0.4–2 items/g	1095 ± 391.2 μm	Fragments, (fibers)	White, (black)	PA, PVC, PP	µRaman	Stroubles Creek and Toms Creek, southwest Virginia, USA	Gray et al., 2024^58^
*F. limosus*	0.37 ± 0.31 items/ind.; 6.13 ± 4.01 items/g	180–2450 μm	Fibers, (films)	Black, (orange, transparent, blue, Green)	PAK (PEST, PU)	µ-FTIR	Lake Maggiore, Piedmont, Italy	This study
*P. clarkii*	0.07 ± 0.06 items/ind.; 0.91 ± 0.79 items/g	220–610 μm	Fibers	Blue, pink	PAK, PEST	µ-FTIR	Lake Maggiore, Piedmont, Italy	This study
*P. leniusculus*	0.20 ± 0.10 items/ind.; 2.41 ± 1.07 items/g	160–1100 μm	Fibers, fragments	Blue, (light blue, black, Transparent, Yellow)	PEST (PE, PVC)	µ-FTIR	Lake Maggiore, Piedmont, Italy	This study

Average abundance of microplastics ± stdev, size, shape, color, polymer, and study sites are reported (values in parentheses represent the least abundant).

P. clarkii = Procambarus clarkii; P. leniusculus = Pacifastacus leniusculus; F. cristavarius = Faxonius cristavarius; F. limosus = Faxonius limosus. EP = epoxy resin; PA = polyamide; PAK = polyacrylate; PE = polyethylene; PEST = polyester; PET = polyethylene terephthalate; PP = polypropylene; PPMA = polymethyl methacrylate; PP: PE = poly (propylene-ethylene) copolymer; PTFE = polytetrafluoroethylene; PU = polyurethane; PVC = polyvinyl chloride.

The present work shows that invasive crayfish exhibit low concentrations of MPs, thus aligning with a recent study by^36^ reporting relatively low levels of plastic contamination in the surface waters of Lake Maggiore, ranging from a minimum of 0.02 plastics/m^3^ to a maximum of 0.29 plastics/m^3^. These levels, originating exclusively from secondary sources, are low compared to those observed in other lakes worldwide^36^.

Our first aim was to evaluate potential species-specific differences in MP uptake. Regarding species-specific content, few information exists on dietary preferences in relation to the coexistence of these three species and how this may influence MP ingestion. Contrary to our expectation (H1), our study did not reveal significant interspecific differences in MP uptake. Among crayfish species, *F. limosus* recorded the highest MP concentration, although this finding was not statistically significant. This higher content may be attributed to its detritivorous feeding habits, especially in competition with more aggressive species. *Faxonius limosus* primarily feeds on detritus from sediments^49^ which are known to accumulate high MP level^12^, potentially explaining the highest concentration recorded in this species^49^. reported the coexistence of *F. limosus* with *P. clarkii* and another invasive crayfish (*P. virginalis*) and observed that the *P. clarkii* occupied a distinct trophic niche. *Procambarus clarkii* showed a more carnivorous behaviour, preying on other crayfish and fish, than *F. limosus*, feeding predominantly on detritus, whereas *P. virginalis* exhibited an intermediate feeding behavior^49^. Similarly, *P. leniusculus* is an omnivore with a generalist diet^60^, whereas *P. clarkii*, although primarily consuming fresh macrophytes and detritus, showed a more pronounced carnivorous habit^61^.

Comparing our MPs concentrations in crayfish with previous studies (Table 1), we found lower concentrations in both *P. clarkii* and *P. leniusculus*, while for *F. limosus*, no comparative data are available. For *P. clarkii*, our analysis revealed lower values compared to those reported in three studies conducted in different ecosystems: two in aquaculture in China^37,59^ and one in Lake Candia in Italy^19^^37^. reported higher MPs concentrations in the digestive tract of crayfish in Shanghai, while^59^ found MPs accumulation in all body parts except the flesh, with significantly higher concentrations in the stomach and gut compared to the gills. The low MP concentrations found in these studies may be due to varying pollution levels influenced by hydrodynamics and plastic usage, with rice-fish culture paddies being isolated by ridges that block microplastic entry^37,59^. Compared to^19^, our values for MPs *per* individual were also lower in the digestive tract and MP content was influenced by crayfish weight, with smaller individuals showing higher MP concentrations, suggesting that feeding habits and metabolic rates could influence MP retention. However, our concentration *per* gram of tissue is higher than that recorded in the same study as well as we did not find any relationships between crayfish size/MP size.

The recorded MP values in *P. leniusculus* are much lower than those reported by^38^ in crayfish gut and tails from watercourses within the Yorkshire Dales National Park. MP concentrations were higher in crayfish guts than in tails, and a positive correlation with MP concentration and urbanization degree was observed^38^. However, our results are more in line with the study by^60^, which found 1–2 items/ind. in crayfish from the Wieprza River in Poland, though our values remain slightly lower. Despite *F. limosus* showing the highest MP concentrations among the species in our study, these values remain lower than those reported for *P. leniusculus* and *P. clarkii*^37,38,59^.

Our findings from Lake Maggiore suggest that crayfish reached similar concentrations of *Perca fluviatilis* (perch; 1.73 ± 1.83 items/ind^62^. , , but much lower than MP concentrations of some sessile taxa, such as bivalves which can reach up to 6 items/ind. in Lake Maggiore^63^, reinforcing the evidence that filter-feeding organisms retain more MPs^16^.

Synthesizing our insights, it seemed that variability in MP contamination depended on the environmental context and the surrounding land use. Differences in MP concentrations could be attributed to factors such as local plastic use and pollution levels^64^. However, to gain a clearer understanding of species-specific uptake differences, further studies should evaluate multiple species coexisting in the same environment to assess potential influences of feeding habits and competition.

### Qualitative characterization of MPs across three invasive coexisting crayfish

Contrary to our hypothesis (H2), we did not find significant differences in MP size distribution across species since most of the MPs were < 1 mm. This finding aligns with previous studies reporting that crayfish predominantly ingest MPs < 1 mm, regardless of species^19,37,58^. These MPs mainly derived from the breakdown of larger plastic debris and are easily ingested by aquatic organisms^16,65^. Additionally, the excretion of MPs by aquatic animals contributed to their redistribution within aquatic ecosystems, potentially increasing their input and further fragmenting them into smaller particles during digestion process^66^.

The lack of correlation between biometric parameters and MP characteristics (size and number of items) may be attributed to the limited sample size that can hinder our results. Other studies have reported no significant relationship between crayfish size and MP size ingested^60^. Interestingly, although not statistically significant, among the studied species, *F. limosus* which shows the smallest size, concentrates more MPs as reported by^19] and [38^ for *P. clarkii*. This last species shows an age-related shift in diet: juveniles feed primarily on macroinvertebrates, which ingest large MP amounts^18^, while adults consume more detritus and plants^67^. This may explain why juveniles ingest more MPs, differing from the usual pattern of higher MP levels at higher trophic levels, both in freshwater and marine environments^19,68^.

Once again contrary to our expectation (H3), the other qualitative characteristics of MPs did not significantly differ across invasive crayfish. Fibers were the most prevalent form, consistent with previous studies both on freshwater biota^16,69^ and on abiotic matrices^12,70^. This prevalence suggests a mainly secondary origin, as fibers, fragments and films, indicative of plastic degradation, were the main shapes detected, while primary MPs, such as pellets, were absent^36^. Regarding color, dark-colored MPs, such as blue and black, were mainly detected, consistent with findings in other invasive crayfish^19,60^; whereas other studies observed mainly white or transparent MPs^37,58,59^. Interestingly^59^, observed that red and transparent MPs were more prevalent in the digestive systems of crayfish (*P. clarkii*) than in the environment. This pattern may be influenced not only by the crayfish’s feeding habits but also by the traits of their prey, as crayfish are more likely to accidentally ingest MPs that resemble their prey in color^59^.

PEST and PAK were the most abundant polymers, alongside other common types such as PE and PVC. In this context, it is important to note that no nylon (polyamide) particles from crayfish traps were observed in analysed specimens (Table S3). Previous studies on invasive crayfish have reported PEST, PE, PP, and PET as the most prevalent polymers^19,37^. Similarly to our findings, other taxa from Lake Maggiore, such as bivalves, have been found to ingest primarily PEST and PAK fibers^63^. MP uptake in aquatic organisms can be also influenced by bioavailability of pollutants, with studies indicating that the shape, color, and polymeric composition of MPs are often similar across various species and abiotic matrices^19,37^.

Lake Maggiore is influenced by multiple environmental pressures from urbanization, industrial activities, and tourism, which contribute to the presence of MPs^71^. Urban expansion has led to increased population and infrastructure demand, resulting in higher waste production^72^. MP diversity in the lake stems from synthetic fibers from laundry^73^, ineffective wastewater treatment^74^, recreational activities, seasonal plastic waste from tourism^36^, and atmospheric deposition^10^.

The exposure and uptake of MPs in crayfish in the range 1 mg/L − 100 mg/L pose toxic effects mainly investigated in laboratory conditions using generally *P. clarkii* and highlight immunological, neurological, oxidative stress, reproductive and developmental effects, as well as microbiome dysbiosis^75,76^. In general, smaller and irregular MPs tend to have more harmful effects on aquatic crustaceans and their impact is further amplified when they coexist with other contaminants^68^. Given the growing evidence of microfiber uptake in aquatic organisms, further research is needed to assess their ecotoxicological impact, which remains understudied compared to other MP shapes, such as the uncommon pellets and beads^77^.

Although our study focuses on invasive crayfish that, by law, should not be commercialized^22,78^, from a human perspective MP presence in crayfish raises significant concerns, as they may act as vectors of heavy metals and pathogens making these species unsafe for consumption^60^. noted that only the edible portions of crayfish, such as the abdomen and the chelae, are typically consumed, while the stomach and the intestines, where MPs are commonly detected, are discarded. However, the potential transfer of MPs and associated contaminants into crayfish muscle tissue remains uncertain, underscoring the need for further research to evaluate possible risks to human health.

## Conclusions

The hydrographic basin of Lake Maggiore plays a key role in the transportation of MPs, collecting runoff from various sources and conveying pollutants into the lake. Given these dynamics, MPs become ubiquitous, affecting different areas of the lake and its aquatic organisms. This makes Lake Maggiore an ideal site for monitoring the combined effects of human activities on freshwater ecosystems and understanding the pathways through which MPs enter and circulate within aquatic environments and organisms.

Our findings indicate no significant interspecific differences in MP concentration or qualitative characteristics across *F. limosus*, *P. leniusculus*, and *P. clarkii*. To better understand whether MP content differences arise from their coexistence and trophic interactions, future studies should assess how species occurrence affects diet under controlled conditions.

Although MP concentrations in crayfish were relatively low, their presence remains concerning due to the potential co-transport of other contaminants through the food web. Most MPs found were PEST and PAK fibers (< 1 mm), suggesting a likely domestic source of plastic pollution, and highlighting the role of household waste and fabric degradation in MPs presence in the lake. Overall, the MP polymeric composition may be due to several anthropogenic sources (urban, industrial, and touristic activities).

Future research should focus on evaluating the broader ecological impacts of MP pollution in the lake, particularly its interactions with other stressors such as additional pollutants, biodiversity loss, and climate change. Developing effective strategies to protect the lake’s ecosystem should remain a priority.

Invasive crayfish hold significant potential as sentinels of MP pollution in dynamic aquatic environments owing to their widespread distribution across various ecosystems. Furthermore, the removal of invasive crayfish for management purposes could remove ingested MPs from the ecosystem, although this should not be interpreted as ecological remediation, as the primary sources of pollution persist and required removal. Future studies on MP accumulation in other organs will help clarify the potential contribution of such management actions.

## Supplementary Information

Below is the link to the electronic supplementary material.

Supplementary Material 1

## Data Availability

Data are available from the corresponding author on reasonable request.
